# Noradrenergic regulation of cue-guided decision making and impulsivity is doubly dissociable across frontal brain regions

**DOI:** 10.1007/s00213-023-06508-2

**Published:** 2023-11-25

**Authors:** Chloe S. Chernoff, Tristan J. Hynes, Jackson D. Schumacher, Shrishti Ramaiah, Dimitrios K. Avramidis, Leili Mortazavi, Stan B. Floresco, Catharine A. Winstanley

**Affiliations:** 1 Graduate Program in Neuroscience, Faculty of Medicine, Djavad Mowafaghian Centre for Brain Health, University of British Columbia, Vancouver, BC Canada; 2 Present Address: Department of Psychology, Downing Site, University of Cambridge, Cambridge, UK; 3 Department of Psychology, Djavad Mowafaghian Centre for Brain Health, University of British Columbia, Vancouver, BC Canada; 4 Present Address: Department of Psychological and Brain Sciences, University of Massachusetts Amherst, Amherst, MA USA; 5 Present Address: Department of Psychology, University of Concordia, Montreal, QC Canada; 6 Department of Psychology, Stanford University, Stanford, CA USA

**Keywords:** Noradrenaline, Prefrontal cortex, Prelimbic cortex, Orbitofrontal cortex, Decision making, Impulsivity, Rat gambling task

## Abstract

**Rationale:**

Win-paired stimuli can promote risk taking in experimental gambling paradigms in both rats and humans. We previously demonstrated that atomoxetine, a noradrenaline reuptake inhibitor, and guanfacine, a selective α2A adrenergic receptor agonist, reduced risk taking on the cued rat gambling task (crGT), a rodent assay of risky choice in which wins are accompanied by salient cues. Both compounds also decreased impulsive premature responding.

**Objective:**

The key neural loci mediating these effects were unknown. The lateral orbitofrontal cortex (lOFC) and the medial prefrontal cortex (mPFC), which are highly implicated in risk assessment, action selection, and impulse control, receive dense noradrenergic innervation. We therefore infused atomoxetine and guanfacine directly into either the lOFC or prelimbic (PrL) mPFC prior to task performance.

**Results:**

When infused into the lOFC, atomoxetine improved decision making score and adaptive lose-shift behaviour in males, but not in females, without altering motor impulsivity. Conversely, intra-PrL atomoxetine improved impulse control in risk preferring animals of both sexes, but did not alter decision making. Guanfacine administered into the PrL, but not lOFC, also altered motor impulsivity in all subjects, though in the opposite direction to atomoxetine.

**Conclusions:**

These data highlight a double dissociation between the behavioural effects of noradrenergic signaling across frontal regions with respect to risky choice and impulsive action. Given that the influence of noradrenergic manipulations on motor impulsivity could depend on baseline risk preference, these data also suggest that the noradrenaline system may function differently in subjects that are susceptible to the risk-promoting lure of win-associated cues.

**Supplementary information:**

The online version contains supplementary material available at 10.1007/s00213-023-06508-2.

## Introduction

The thrilling lights and buzzers of a modern casino may have a greater impact on the development of problematic gambling behaviour than once thought. When light and sound cues are paired with wins in experimental gambling tasks, a larger proportion of both rodent and human subjects adopt risky decision making strategies (Barrus & Winstanley 2016; Cherkasova et al. 2018; Spetch et al. 2020). The neurobiology behind cue-exacerbated risk preference, however, has not been wholly elucidated (Winstanley & Hynes 2021). Gambling-related cues enhance arousal (Dixon et al. 2014) and can trigger cravings to gamble (Park et al. 2015; Potenza et al. 2003). Those with gambling disorder (GD) demonstrate heightened attentional biases to such cues (McGrath et al. 2018; van Holst et al. 2012). Such susceptibility to the allure of cues may contribute to the propensity toward maladaptive states of altered consciousness and attention (Tricker et al. 2016), such as “the zone” or “dark flow,” wherein individuals with GD exhibit trance-like game immersion. In these states, subjects become insensitive to non-game related stimuli, such as passing time and personal life stressors (Dixon et al. 2019; Schull 2005). Noradrenaline, as the main regulator of attention and arousal in the central nervous system, is therefore important to consider when examining the mechanisms behind the risk promoting effects of win-paired stimuli.

We previously showed that systemic administration of a noradrenaline transporter (NET) blocker, atomoxetine, and an α2 adrenergic receptor agonist, guanfacine, improved decision making on the cued rat gambling task (crGT) by shifting preference away from highly cued, high-risk high-reward options toward safer options that yield more consistent smaller wins (Chernoff et al. 2021). This is in stark contrast to data from the uncued version of the rat gambling task in which atomoxetine had no effect or even slightly reduced optimal choice (Baarendse et al. 2014; Silveira et al. 2016). Noradrenaline may therefore uniquely modulate decisions that are made under the influence of risk-promoting win-concurrent cues. Both atomoxetine and guanfacine were also able to reduce impulsive responses made prematurely on the crGT (Chernoff et al. 2021). Noradrenaline signaling in the prefrontal cortex, particularly at postsynaptic α2 receptors, is thought to underly the beneficial effects of noradrenergic compounds on working memory and attention (Arnsten et al. 1988, 1996; Cai et al. 1993). Whether noradrenaline likewise acts in the frontal cortices to alter decision making and impulse control on the crGT has yet to be tested.

Notably, areas of the prefrontal cortex such as the orbitofrontal cortex (OFC) and medial prefrontal cortex (mPFC) receive discrete and direct projections from the noradrenergic locus coeruleus (LC) (Agster et al. 2013; Chandler et al. 2013, 2014). These frontal areas are also functionally heterogeneous. The OFC is important for establishing, updating, and using cue- and action-outcome associations to guide optimal decision making (reviewed in Bechara et al. 2000; Izquierdo 2017; Schoenbaum et al. 2009). Recent evidence indicates that noradrenaline in the OFC, but not mPFC, is necessary for appropriately updating action-outcome representations to guide subsequent decisions (Cerpa et al. 2022), specifically implicating noradrenaline in OFC-dependent value assessment. Whereas OFC neurons tracked the utility of an option as it changed across a session, mPFC neuron activity instead consistently responded to small immediate rewards (Hong et al. 2019). Additionally, Bari et al. demonstrate that the noradrenergic LC exerts divergent control over sustained attention and inhibitory control via afferents projecting to the dorsal mPFC and ventral lOFC, respectively (2020).

The prelimbic cortex (PrL) of the mPFC is strongly implicated in response inhibition and action selection (Feja & Koch 2014; Narayanan et al. 2006), with multiple studies demonstrating a significant relationship between PrL neuron activity and motor impulsivity (Hardung et al. 2017; Hayton et al. 2011; Moschak & Carelli 2021; Narayanan et al. 2006; Narayanan & Laubach 2006). Noradrenaline itself is known to be a potent regulator of impulsive action (Bari et al. 2009; Chamberlain & Sahakian 2007; Robinson et al. 2008), with the anti-impulsivity properties of noradrenergic drugs like atomoxetine well-established in the literature (Bari et al. 2009; Economidou et al. 2012; Navarra et al. 2008; Robinson et al. 2008), and further corroborated by rGT data from our laboratory (Chernoff et al. 2021; Silveira et al. 2016). Yet, it is unknown whether noradrenaline modulates impulse control on the crGT through direct action in frontal areas such as the PrL.

Given our previous findings that noradrenergic manipulations significantly influence cue-guided gambling-like behaviour in rats, we aimed to discern the prefrontal substrates which may orchestrate noradrenaline’s contributions to risk taking and motor impulsivity in the presence of win-paired cues. Here, we pharmacologically manipulated local noradrenaline signaling in the lateral OFC (lOFC) or PrL as rats performed the crGT. Considering the dissociable roles of the lOFC and PrL in risk assessment and impulse control, we predicted that pharmacologically enhancing noradrenaline signaling in the lOFC would promote safer decision making, while intra-PrL noradrenergic manipulations would reduce impulsive premature responses.

## Methods

### Subjects

In total, 32 male and 33 female Long Evans rats (Charles River Laboratories, St. Constant, QC) were used for the behavioural experiments, divided into four groups based on sex and targeted brain region (16 male-lOFC, 16 female-lOFC, 16 male-PrL and 17 female-PrL). Rats were pair- or trio-housed with same-sex cagemates in a climate-controlled colony room on a reverse 12-h light–dark cycle (lights off at 08:00am; temperature 21 °C). At least one week prior to the start of behavioral training, animals were food restricted to ~ 85% of their free feeding weight and maintained at ~ 15 g of standard rat chow per day for males and ~ 11 g per day for females. Water was available ad libitum in the homecage. For days on which behavioural testing or training occurred, rats were fed directly following the behavioural session. All housing conditions and testing procedures were in accordance with the guidelines of the Canadian Council of Animal Care, and all protocols were approved by the Animal Care Committee of the University of British Columbia, Vancouver.

### Apparatus

Behavioral testing was conducted in 32 identical five-hole operant chambers (30.5 × 24 × 21 cm; Med Associates, St. Albans, VT, USA), each enclosed in a ventilated sound-attenuating cabinet (Med Associates, St. Albans, VT, USA). Boxes were equipped with fans for air circulation and extrinsic noise cancellation. Along the curved wall of the chamber was an array of five nose-poke holes, each equipped with an infrared detector and a yellow LED stimulus light. On the opposite wall, a food tray was positioned to deliver dustless sugar-coated food pellets (45 mg, Formula P, Bio-Serv, Frenchtown, NJ, USA). The chamber was illuminated by a white house light attached to the roof. Apparatus control and data collection were conducted using code written by CAW in MEDPC (Med Associates) running on standard IBM-compatible computers.

### Cued rat gambling task (crGT)

**Fig. 1:**
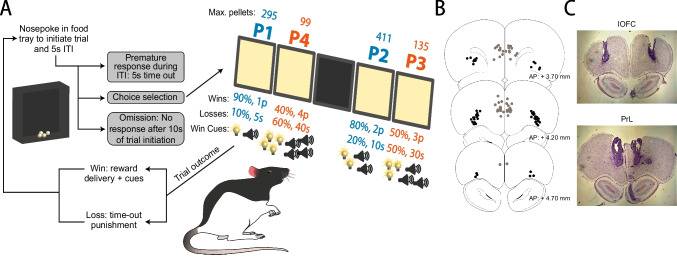
Schematic of the cued rat gambling task (crGT) and accepted cannula placements. **A** In the crGT, rats sample from four nosepoke holes associated with varying probabilities and magnitudes of sugar pellet wins and time-out punishments. A trial is initiated by a nosepoke in the food tray (left side of the diagram), following which the four options are illuminated and the rat is free to make a choice. The probabilities (listed in % likelihood) and magnitudes (listed in sugar pellets won or duration of time-out in seconds) of wins and losses are listed for each of the optimal (P1 and P2) and risky (P3 and P4) options. On winning trials, sugar pellet rewards are accompanied by audiovisual cues that scale in complexity with win magnitude, such that the riskiest options are associated with the most complex cues (see Barrus & Winstanley 2016 for audiovisual cue specifications). As a measure of motor impulsivity, premature responses are recorded when the subject responds during the intertrial interval (ITI) during which none of the options are illuminated and a choice cannot be made, and were punished with a 5-s time-out. Omissions were defined as failure to respond at one of the four options within 10 s of trial initiation. **B** Cannula placements were determined from Cresyl stained brain sections, and those appropriately targeting the intended region were plotted onto images adapted from the Rat Brain Atlas (Paxinos & Watson 1998). Black circles indicate lOFC placements while grey circles correspond to PrL cannula placements. Subjects with inaccurate cannula placements (not shown) were excluded from the analyses. **C** Representative Cresyl stained coronal sections for lOFC and PrL cannulae, respectively

As described previously, behavioural training began with two daily 30-min chamber habituation sessions followed by basic operant nose-poke training and seven days of 30-min forced choice crGT sessions, during which rats are exposed to the contingencies of each task option (Barrus & Winstanley 2016; Ferland et al. 2019; Zeeb et al. 2009). Rats then went on to perform the full, 30-min free choice version of the crGT during which they sampled from four of the response openings (the middle hole of the 5-hole array is not used in the crGT). In brief, a nose-poke into the illuminated food tray started each trial and initiated a five second inter-trial interval (ITI), after which the stimulus lights in each of the four response holes lit up. A choice could then be made by a nosepoke into any one of the four illuminated apertures. Each option was associated with a unique magnitude and probability of both a sugar pellet reward and time-out punishment (Fig. 1). During the time-out punishments, the light in the chosen hole flashed slowly at 0.5 Hz, and subjects were unable to initiate a new trial. At the end of each time-out, the food tray was illuminated, and a nosepoke in the food tray was required to start the next trial. The optimal strategy on the crGT is to favor the options that result in a smaller per-trial reward coupled with shorter time-out punishments (P1 and P2). These “safe” options earned the most reward throughout the task due to more consistent wins, less frequent punishment, and shorter time-out penalties compared to the “risky” options (P3 and P4) that delivered larger, uncertain rewards and longer, more frequent punishments. In the crGT, audiovisual cues were concurrently presented with sugar pellet rewards on winning trials. The cues increased in complexity with the magnitude of the reward, similar to the human gambling experience (Barrus & Winstanley 2016).

Much like in the five-choice serial reaction time task, premature responses were used as a measure of motor impulsivity, and were defined as nose poke responses made at the aperture array during the five second ITI. A premature response resulted in a time-out punishment of 5 s, during which the houselight was illuminated. An omission was recorded if the rat failed to respond at one of the four nosepoke options within 10 s of their illumination.

Rats were trained on the crGT 5–7 days a week until performance on all behavioural measures (described below) were deemed statistically stable over the last five consecutive sessions, meaning a repeated measures ANOVA revealed no significant interactions or main effect of session. Behavioural stability was assessed for each cohort of animals separately, and was achieved after 32–43 free choice crGT sessions, depending on the cohort.

## Surgery

After stable baseline crGT performance was attained, animals underwent stereotaxic surgery under isoflurane anesthesia (5% induction; 2% maintenance) to implant beveled 23-gauge stainless steel guide cannulae bilaterally into either the lOFC (*n* = 16 males, 16 females; lOFC: AP =  + 3.5 mm from bregma, ML =  ± 2.6 mm from midline, DV =  − 2.9 mm from dura) or PrL (n = 16 males, 17 females; PrL: AP =  + 3.0 mm from bregma, ML =  ± 0.7 mm from midline, DV =  − 2.8 mm from dura). Guide cannulae were secured to the skull through the aid of four stainless steel screws and a dental acrylic headcap. Sterile 30-gauge obdurators flush with the end of the cannulae were inserted and replaced as necessary throughout the duration of the experiment. Appropriate surgical post-care procedures were followed and animals were given at least 1 week of post-operative recovery in the homecage, with ad libitum food supply, before resuming any behavioural procedures.

### Intracerebral microinfusions

Following post-surgical recovery, animals were reintroduced to the task with 5–8 free choice crGT sessions, to re-establish statistically stable performance. Animals were then habituated to the microinfusion process with a mock infusion, during which sterilized 30-gauge injectors were inserted into the guide cannula and left in place for two minutes. No drug was infused. Animals were then left in the operant box for ten minutes following the mock infusion, prior to performing a crGT session.

Two days after the mock infusion, rats began a series of acute drug challenges with atomoxetine (ATX: 1.5 μg/side, 5.0 μg/ side, saline vehicle) and guanfacine (GFC: 0.005 μg/side, 3 μg/side, saline vehicle). Doses for intracerebral microinfusions were determined based on previous behavioural experiments (Bari et al. 2011; Economidou et al. 2012; Pardey et al. 2013). Atomoxetine hydrochloride and guanfacine hydrochloride were purchased from Sigma-Aldrich (Oakville, Canada). Drug doses were calculated as the salt and dissolved in sterile 0.9% saline. Atomoxetine was infused as a 11 mM or 39 mM solution for low and high doses, respectively, while low and high doses of guanfacine were administered using 41 µM and 24 mM solutions, respectively. Each rat received a total of six infusions: low dose, high dose, and vehicle for both atomoxetine and guanfacine. Each drug was microinfused following a balanced Latin square design (doses: ABC, BCA, CAB; Cardinal and Aitken 2013). Every subject first received either atomoxetine or guanfacine, with drug order counterbalanced across subjects, followed by at least one week of washout prior to beginning a second Latin square for the other compound to mitigate any potential carryover effects.

Drug administration followed a 3-day cycle, starting with a baseline, drug-free crGT session. The following day, animals were dosed and tested on the crGT. Bilateral microinfusions of 0.5 μL per hemisphere were administered at a rate of 0.5 μL/min (for a total infusion time of 1 min) via 30-gauge injector tips that extended 0.8 mm beyond the guide cannulae. Injectors were left in place for an additional minute to allow for diffusion. Following the diffusion period, injectors were removed, sterile obdurators replaced, and animals were placed in the operant chambers for 10 min prior to beginning the crGT (Bari et al. 2011; Economidou et al. 2012; Yates et al. 2016). Animals were not tested nor dosed on the third day of the Latin square schedule.

### Histology

Following completion of all behavioral testing, animals were anesthetized with isoflurane and euthanized by acute carbon dioxide exposure. Brains were immediately extracted and fixed in 4% phosphate buffered formaldehyde for 24–48 h before being transferred to a cryoprotective 30% sucrose and 0.02% sodium azide solution. They were then frozen and sliced into 40-μm coronal sections. Frontal brain sections were stained with cresyl violet for visualization on the Zeiss Axioscan 7 slide scanner (Zeiss, Oberkochen, Germany). Brightfield images were captured at 10 × magnification to confirm cannula placement, and the projected locations of the injector tips protruding from the guide cannulae were mapped onto standard sections adapted from the Rat Brain Atlas (Paxinos & Watson 1998). Rats were excluded from the analyses if their cannulae were misplaced or did not accurately target the prefrontal region of interest as defined by the Rat Brain Atlas (Paxinos & Watson 1998).

### Behavioural measures and data analysis

All statistical analyses were completed using SPSS Statistics 27.0 software (IBM, Chicago, IL, USA). As per previous reports, the following main crGT variables were analyzed: percentage choice of each option (number of times option was chosen/total number of choices × 100), decision making score (calculated using percent choice variables, i.e., score = [(P1 + P2) − (P3 + P4)]), and percentage of premature responses (number of premature responses/total number of trials initiated × 100). Other crGT behavioural variables including the sum of omitted responses, sum of trials completed, and average latencies to choose an option and collect reward were also analyzed. Variables that were expressed as a percentage or proportion were subjected to an arcsine transformation prior to statistical analysis to limit the effect of an artificially imposed ceiling (i.e., 100%). The last five post-surgery crGT sessions that were statistically stable (i.e., a repeated-measures ANOVA in which neither the main effect of session or the session × choice interaction were not significant) were used to determine baseline performance. Animals with mean positive (i.e., ≥ 0) baseline decision making scores were designated as “optimal performing” (OPT) subjects, whereas rats with negative risk scores at baseline were classified as “risk-preferring” (RP). If a two-way repeated measures ANOVA with dose (three levels: vehicle, low dose and high dose) and choice (four levels: P1, P2, P3, and P4) as within-subjects factors came out with a significant interaction or main effect of choice, individual options were subject to separate repeated measures analysis. All variables were analyzed using a repeated measures ANOVA with dose (or session in the case of baseline data) as a within-subjects factor, and both sex and risk preference as between-subjects factors. If sphericity was violated as determined by Mauchley's test of sphericity, a Huynh–Feldt correction was applied.

Results were considered statistically significant if* p*-values were less than or equal to α = 0.05. Any main effects or interactions of significance resulting from the repeated measures ANOVA were further analyzed via one-tailed paired samples t-tests with a Bonferroni correction applied for the number of comparisons made, given the directionality predicted based on the graphical ANOVA output. To determine if there was an effect of cannulation surgery on crGT behaviour, averaged data from the last five stable sessions of pre- and post-surgery baseline were compared using repeated measures analyses as described above. All data were plotted as mean ± SEM. For within-subjects analyses, error bars were corrected as described previously to prevent overestimation of standard error and to more accurately depict within-subjects variation (Betts et al. 2021).

To investigate how our pharmacological manipulations may have influenced the tendency to switch choice strategies following a certain task outcome, we performed trial-by-trial analyses on the Latin square data (Mortazavi et al. 2023). For each trial, behaviour was classified as either a win-stay or lose-shift depending on the choice category (safe: P1/P2, or risky: P3/P4) and outcome (win or loss) of the previous trial, and whether the animal chose from the same choice category on the following trial. If an animal chose one of the options within a choice category on the previous trial, then chose the other option from within the same choice category, this counted as a “stay” (i.e., choosing safe P1 on the first trial, then safe P2 on the next trial). The trial-by-trial behaviour was further classified by the magnitude of reward such that outcomes from safe choices were labeled small wins and small losses, and outcomes following risky choices were considered big wins and big losses. Trial-by-trial switching behaviour was expressed as a proportion of total completed trials, and the resultant proportion data were subject to repeated measures analyses as described above. Trials during which the subject made an omission or premature response were excluded from trial-by-trial analyses.

## Results

### Histology

Cresyl violet staining was used to confirm cannula placements in either the lOFC or PrL cortex, as defined by Paxinos and Watson (1998). Acceptable projected infusion sites were plotted onto coronal sections adapted from the Rat Brain Atlas (Fig. 1B; Paxinos & Watson 1998). Representative micrographs of proper cannula placements are provided in Fig. 1C. One lOFC male and two PrL females were excluded from subsequent analyses due to misplaced cannulae that were ventral to the respective targets. One lOFC male, two PrL males, and two PrL females were excluded from analyses due to obstructed, bent, or damaged cannulae that precluded completion of all six microinfusions. One lOFC male died during surgery, and two lOFC females were euthanized prior to undergoing microinfusions due to poor health. The total number of rats in each group that were included in analyses were as follows: 13 male-lOFC, 14 female-lOFC, 14 male-PrL, and 13 female-PrL.

### Baseline crGT behaviour

**Fig. 2:**
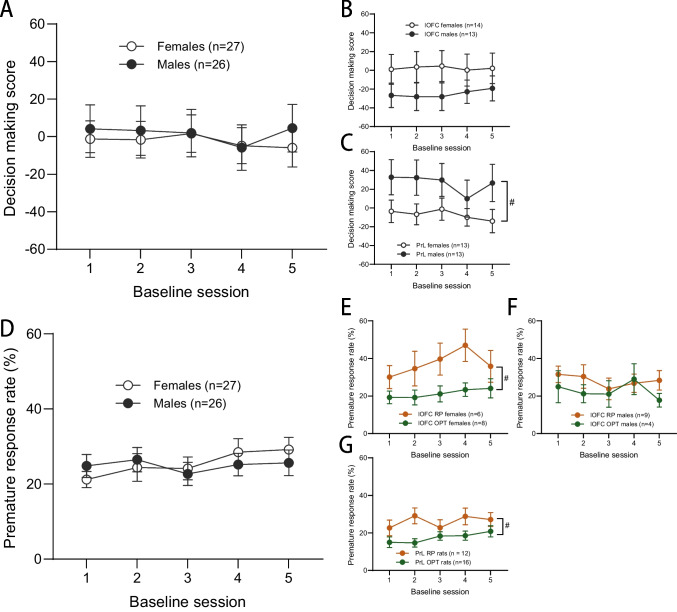
Baseline crGT behaviour. Decision making score did not significantly differ between sexes **A** overall, nor **B** in the lOFC cohort when considered separately, yet **C** PrL males demonstrated higher decision making scores than PrL females at baseline. **D** Regarding baseline motor impulsivity, there was no overall sex difference. **E** Risk preferring (RP) females in the lOFC cohort made more premature responses than optimal performing (OPT) females, yet **F** RP and OPT males in the lOFC cohort did not differ in premature responding. **G** RP rats in the PrL cohort, irrespective of sex, made more prematures responses than OPT PrL rats. Data are mean ± SEM. #* p* < 0.05 between subjects effect

Stable pre- and post-surgical data were compared to screen for any potential effects of cannulation surgery on crGT performance. No significant differences were found for any behavioural variable in any of the cohorts (all Fs < 2.415, ps > 0.076), indicating that the indwelling cannulae and/or surgery did not impact behaviour. Baseline performance was determined from the last five statistically stable post-surgery crGT sessions. Overall, there was no significant effect of sex on baseline decision making score (Fig. 2A; sex: F_1,46_ = 0.618, *p* = 0.436). However, given that data from each cohort could potentially be analyzed independently, we compared baseline performance measures between rats that underwent intra-lOFC or intra-PrL cannulation. Decision making scores of male and female rats in the lOFC cohort did not significantly differ (Fig. 2B; sex: F_1,23_ = 1.882, *p* = 0.083), yet PrL males demonstrated more optimal decision making scores at baseline than PrL females (Fig. 2C; sex: F_1,24_ = 4.597, *p* = 0.042). This was consistent with a pre-surgical baseline sex difference observed in score in the PrL cohort only (PrL- sex: F_1,26_ = 4.391, *p* = 0.046; lOFC- sex: F_1,25_ = 2.393, *p* = 0.134) across the last five stable days of training prior to surgery. This significant difference between males and females in this cohort likely reflects the small number of subjects and the sizeable between-subjects variation in score possible on the cued rGT. Cohorts were not run concurrently, therefore matching subgroups for baseline behavior was unfortunately not possible. As expected, there was a significant between-subjects effect of risk preference on score in both cohorts (lOFC: F_1,23_ = 82.945,* p* < 0.001; PrL: F_1,24_ = 55.402,* p* < 0.001). Individual choice options were analyzed using a two-way ANOVA with choice and session as within-subjects factors. No significant interactions with choice were found for either lOFC or PrL animals (all Fs < 3.210, ps > 0.085), and as such subsequent analysis of individual options were not conducted.

Regarding baseline impulsive premature responses, no overall sex differences were found (Fig. 2D; F_1,46_ = 0.030,* p* = 0.863). Risky females in the lOFC cohort made more impulsive premature responses than their optimal performing counterparts (Fig. 2E; females- risk preference: F_1,12_ = 4.873,* p* = 0.047), yet impulsivity did not differ with risk preference in male lOFC rats (Fig. 2F; sex × risk preference: F_1,23_ = 6.787,* p* = 0.016; males- risk preference: F_1,11_ = 2.261,* p* = 0.161). Risk preferring rats in the PrL cohort, irrespective of sex, exhibited higher impulsivity (Fig. 2G; risk preference: F_1,24_ = 4.420,* p* = 0.046). Further, females took longer to both make a choice and collect food reward than males, yet only in the PrL cohort (Figure S1 A-D; PrL- collect latency: F_1,24_ = 7.865,* p* = 0.010; choice latency: F_1,24_ = 5.132,* p* = 0.033; lOFC- all Fs < 3.129,* p* = 0.090). lOFC females completed more trials than lOFC males (Figure S1 E; sex: F_1,23_ = 5.812,* p* = 0.024) while PrL males completed more crGT trials than PrL females (Figure S1 F; sex: F_1,24_ = 15.755,* p* < 0.001), consistent with the directionality of observed differences in baseline decision making scores in both cohorts. There were no significant differences in omitted trials in either group (Figure S1 G&H; all Fs < 2.355, ps > 0.139).

### Behavioural effects of drug infusions into the lOFC

#### Atomoxetine

##### Decision making

**Fig. 3:**
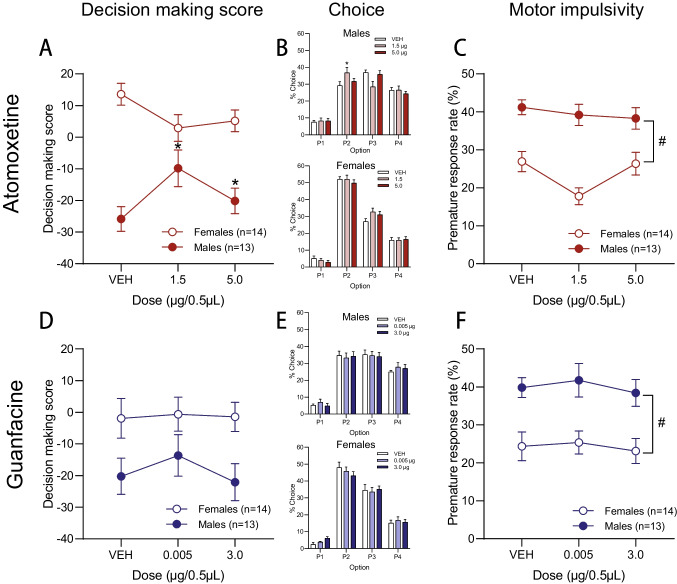
Behavioural effects of intra-lOFC drug infusions on crGT performance. **A** Both doses of atomoxetine improved decision making score in male rats when infused into the lOFC, yet intra-lOFC atomoxetine was unable to significantly influence score in females. **B** The low dose of atomoxetine enhanced preference for the most advantageous option P2, driven by a significant increase in P2 choice in OPT males, and produced a trend-level decrement in choice of the risky option P3. No significant changes in choice were observed for females. **C** Intra-lOFC atomoxetine did not precipitate significant changes in impulsive premature responses in either sex, yet males demonstrated higher impulsivity than females overall. **D** Guanfacine did not influence decision making score nor **B** premature responding when infused into the lOFC, yet males demonstrated higher premature responding than females. * *p* < 0.05 compared to VEH. &* p* < 0.08 main effect of dose. # *p* < 0.05 between-subjects effect. Data are presented as mean ± within-subjects corrected SEM

Intra-lOFC atomoxetine increased decision making score selectively in male rats (Fig. 3A; dose × sex: F_2,46_ = 6.392,* p* = 0.005; females- dose; F_2,24_ = 2.392,* p* = 0.113; males- dose: F_2,22_ = 4.109,* p* = 0.030; 1.5 µg vs VEH: t_12_ = -1.879,* p* = 0.042; 5.0 µg vs VEH: t_12_ = -1.823,* p* = 0.047). There were no significant interactions with risk preference (all Fs < 2.360, ps > 0.111). As such, optimal performing and risk preferring rats of each sex were collapsed for subsequent analyses. It appeared as if males generally had lower decision making scores than females, although the between-subjects sex difference did not reach statistical significance (sex: F_1,23_ = 0.248,* p* = 0.623). To explore the possibility that the sex-specific effect of lOFC atomoxetine on score may have been partially driven by the subjectively higher risk preference demonstrated by our lOFC males, baseline risk score was covaried in the above repeated measures ANOVA. The dose × sex interaction (F_2,44_ = 6.462,* p* = 0.003) and main effect of dose in males remained significant when controlling for baseline score (males: F_2,20_ = 3.609,* p* = 0.046; females: F_2,22_ = 1.802,* p* = 0.188), validating that the sex-specific effect was not driven by higher levels of risk preference in males. An omnibus analysis revealed that atomoxetine influenced choice of individual options in males only (choice × dose × sex: F_6,138_ = 4.872,* p* = 0.003; choice × dose- males: F_6,66_ = 3.014,* p* = 0.029; females: F_6,72_ = 1.185,* p* = 0.324). As such, the individual choice options were further analyzed for males only. The lower dose of atomoxetine increased choice of the best option, P2, in optimal decision-makers (Fig. 3B; dose × risk preference: F_2,22_ = 4.228,* p* = 0.028; OPT males- 1.5 µg vs VEH: t_3_ = -2.539,* p* = 0.042; all other Fs < 0.921, ps > 0.212), and appeared to reduce choice of the risky option P3 in all males, yet this effect did not reach statistical significance (Fig. 3B; dose: F_2,22_ = 2.975,* p* = 0.072). No changes were observed in choice of the safe option P1 nor the riskiest option P4 (Fig. 3B; all Fs < 2.622, ps > 0.095).

##### Impulsivity

Neither dose of atomoxetine influenced impulsive premature responding when infused into the lOFC (Fig. 3C; dose: F_2,46_ = 2.675,* p* = 0.080). Visual inspection of the data indicated that females may be more susceptible to the subthreshold anti-impulsivity effect of atomoxetine infused into the lOFC, yet the ANOVA yielded no significant interactions with sex (all Fs < 0.930, ps > 0.402). There was, however, a significant between-subjects effect of sex whereby males exhibited greater motor impulsivity than females (Fig. 3C; sex: F_1,23_ = 9.253,* p* = 0.006). No other significant main effects or interactions were reported (all Fs < 1.829, ps > 0.189).

##### Other behavioural variables

Intra-lOFC atomoxetine did not significantly affect latency to collect food pellet rewards in either sex, despite a significant dose × sex interaction (Figure S2; dose × sex: F_2,46_ = 3.909,* p* = 0.037; dose- males: F_2,22_ = 2.344,* p* = 0.119; females: F_2,24_ = 1.748,* p* = 0.203). No significant effects or interactions were noted for trials completed, omissions, or choice latency (Figure S2; all Fs < 2.310, ps > 0.142).

#### Guanfacine

##### Decision making

Intra-lOFC guanfacine did not significantly impact decision making score (Figs. 3D; all main effects and interactions: all Fs < 0.791, ps > 0.460), nor did it influence choice of individual options (Fig. 3E; all Fs < 1.546, ps > 0.183).

##### Impulsivity

There was no main effect of, or interactions with, dose of intra-lOFC guanfacine on premature responses (Fig. 3F; all Fs < 2.353, ps > 0.107). There was, however, a between-subjects effect of sex wherein males made more impulsive premature responses than females (Fig. 3F; sex: F_1,22_ = 6.424,* p* = 0.019).

##### Other behavioural variables

No significant main effects or interactions were detected for trials completed, omissions, and latencies to choose and option or collect reward (Figure S2; all Fs < 1.176, ps > 0.318).

### Behavioural effects of drug infusions into the PrL

#### Atomoxetine

##### Decision making

**Fig. 4:**
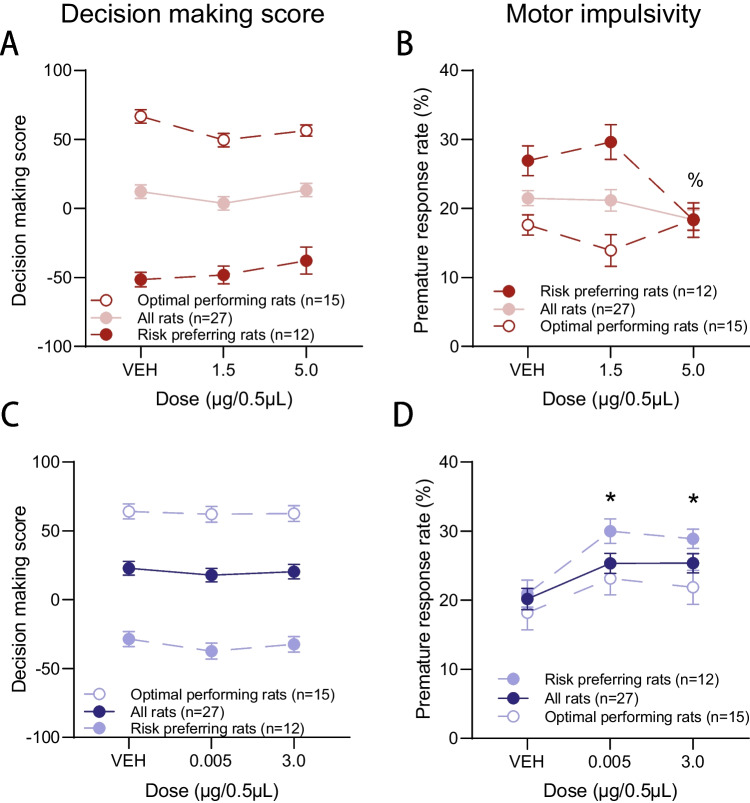
Behavioural effects of intra-PrL drug infusions*.* **A** Atomoxetine infused into the PrL did not alter decision making score, yet **B** the highest dose of intra-PrL atomoxetine selective reduced premature responding in RP rats. **C** Guanfacine did not alter decision making score when infused into the PrL. **D** Intra-PrL guanfacine increased premature responses in all rats at both doses. %* p* < 0.05 compared to VEH in RP group only. * *p* < 0.05 compared to VEH. Data are presented as mean ± within-subjects corrected SEM

Atomoxetine infused into the PrL did not significantly influence decision making score (Fig. 4A; all Fs < 2.466, ps > 0.098). Our omnibus choice ANOVA also did not reveal any significant interactions between dose and choice (all Fs < 2.205, ps > 0.081), and therefore individual options were not separately analyzed.

##### Impulsivity

Atomoxetine reduced impulsive premature responses when microinfused into the PrL, yet this effect was only observed in risk preferring animals following the high dose (Fig. 4B; dose × risk preference: F_2,44_ = 5.760,* p* = 0.006; RP rats- dose: F_2,20_ = 4.635,* p* = 0.022; 5.0 µg vs VEH: t_11_ = 2.545,* p* = 0.014; OPT rats- dose: F_2,24_ = 1.525,* p* = 0.238). In the interest of transparency, it appeared that risk preferring rats were more impulsive than optimal performers following vehicle infusions, suggesting that this selective effect on premature responding in risky rats may have been driven by high baseline impulsivity. To test whether this effect was driven by risk preference, baseline decision making score was covaried in the initial repeated measures analysis. With score as a covariate, intra-PrL atomoxetine no longer impacted premature responding (dose- RP rats: F_2,18_ = 1.640,* p* = 0.222; OPT rats: F_2,22_ = 0.086,* p* = 0.918), confirming that the observed selective reduction in impulsivity was related to high baseline risk preference.

##### Other behavioural variables

Repeated measures ANOVAs revealed no significant main effects of, or interactions with, atomoxetine dose for trials completed, omissions, reward collection latency, or choice latency (Figure S4; all Fs < 2.179, ps > 0.136).

#### Guanfacine

##### Decision making

Guanfacine did not significantly change score when administered into the PrL, even though the ANOVA indicated a significant dose × sex × risk preference interaction (Fig. 4C and S3 B; dose × sex × risk preference: F_2,46_ = 3.728,* p* = 0.032; dose- OPT females: F_2,10_ = 0.392,* p* = 0.686; RP females: F_2,12_ = 0.258,* p* = 0.777; OPT males: F_2,16_ = 0.280,* p* = 0.672; RP males: F_2,8_ = 3.227,* p* = 0.146). An omnibus analysis revealed a significant choice × dose × sex × risk preference interaction (F_6,138_ = 2.612,* p* = 0.020), yet further analyses of individual options revealed that guanfacine was unable to significantly influence choice in any of four options when compared to vehicle infusions (all Fs < 3.022, ps > 0.110).

##### Impulsivity

Both doses of intra-PrL guanfacine significantly increased the rate of impulsive premature responding in all rats compared to vehicle (Fig. 4D; dose: F = 4.436,* p* = 0.017; 0.005 µg vs VEH: t_26_ = 2.415,* p* = 0.012; 3.0 µg vs VEH: t_26_ = 2.344,* p* = 0.013).

##### Other behavioural variables

No main effects or interactions were noted for omitted trials, choice latency, reward collection latency, or trials completed (Figure S4; all Fs < 2.098, ps > 0.161).

### Trial-by-trial analyses

**Fig. 5:**
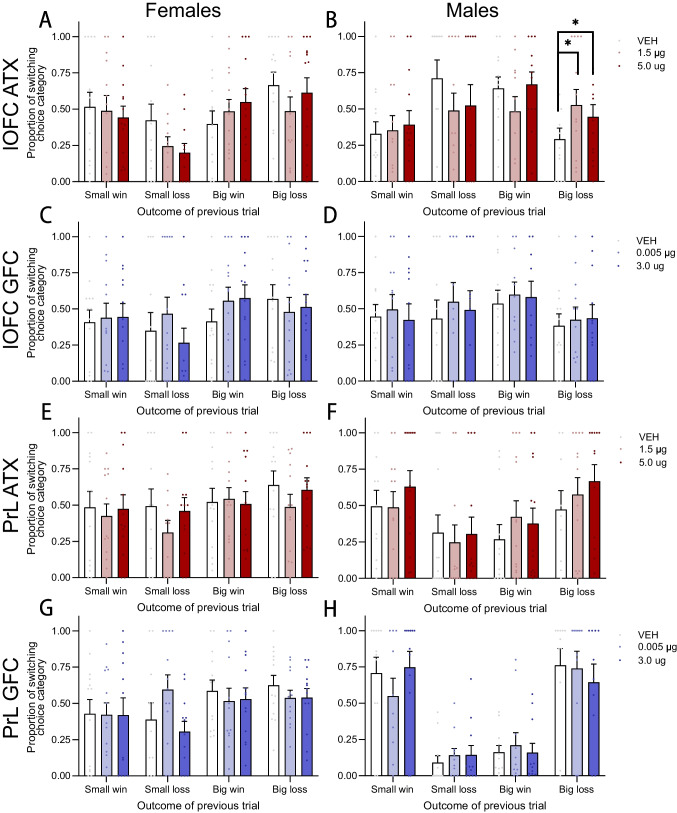
Effects of all pharmacological manipulations on win-stay lose-shift behaviour*.* **A** Infusions of atomoxetine into the lOFC did not influence shifting behaviour in females, but **B** both doses of atomoxetine increased the tendency of males to shift choice categories following a large, risky loss. **C**, **D** Intra-lOFC guanfacine had no influence over trial-by-trial behaviour in either sex, and **E**, **H** neither atomoxetine nor guanfacine altered shifting behaviour when infused into the PrL. * p < 0.05 compared to VEH. Data are presented as mean ± within-subjects corrected SEM. Individual data points represent a data point from a single subject

To gain insight into the behavioral mechanisms through which intra-lOFC atomoxetine changed risk preference in male rats, we tested whether subjects behaved differently following a reward or loss outcome. We hypothesized that NET blockade in the lOFC may improve decision making by enhancing reward- and value-related signal-to-noise ratio, thereby increasing win-stay tendency following safe wins, or by promoting flexible responding to punishment, increasing lose-shifts after risky losses. Atomoxetine, when infused into the lOFC, altered trial-by-trial behaviour in male rats only (outcome × dose × sex: F_6,102_ = 3.118,* p* = 0.008; males- outcome × dose: F_6,48_ = 3.122,* p* = 0.019; females: all Fs < 1.145, ps > 0.349). The low dose of atomoxetine increased the proportion of trials on which male rats switched choice categories immediately following a big loss (Fig. 5B; dose: F_2,22_ = 3.939,* p* = 0.034; 1.5 µg vs VEH: t_11_ = 2.352,* p* = 0.019; 5.0 µg vs VEH: t_11_ = 1.785,* p* = 0.051). Choice behaviour following any other task outcome was not significantly affected by intra-lOFC atomoxetine (all Fs < 3.486,* p* > 0.089).

Intra-lOFC guanfacine did not significantly change trial-by-trial behaviour in any group, nor did microinfusions of either atomoxetine or guanfacine into the PrL (Fig. 5 C-H; all Fs < 2.349, ps > 0.114). There was, however, a significant outcome × sex interaction in the prelimbic cohort whereby PrL males generally made fewer switches than females on trials following a small loss or a big win (Fig. 5 E–H; outcome × sex: F_3,42_ = 3.431,* p* = 0.029; small loss- males vs females: t_22_ = 2.372,* p* = 0.014; big win- males vs females: t_22_ = 4.537,* p* < 0.001; all other ts < 1.178, ps > 0.126).

## Discussion

Using local pharmacological manipulations, we revealed the previously undefined role of prefrontal noradrenaline signaling in cue-guided risk taking and impulsivity. These data present a double dissociation such that noradrenergic action in the lOFC influenced decision making in a sex-specific manner, while PrL noradrenaline signaling was a more potent regulator of impulsive action. Specifically, we show that the selective NET inhibitor atomoxetine improved decision making in male rats when microinfused into the lOFC, increasing choice of the most fruitful option P2 in optimal performers, while decision making in females was relatively immune to the beneficial effects of intra-lOFC NET blockade. Consistent with this sex-specific decision making effect, intra-lOFC atomoxetine was also able to improve adaptive switching behaviour following big losses in males only. Atomoxetine in the lOFC did not influence premature responding, a measure of motor impulsivity, and guanfacine, an α2A receptor agonist, had no behavioural effects when infused into the lOFC. In contrast, when administered into the PrL neither noradrenergic compound altered decision making, yet the drugs produced divergent effects on impulse control. A high dose of intra-PrL atomoxetine selectively improved impulse control in risk preferring rats, while both doses of intra-PrL guanfacine conversely enhanced impulsive premature responding in all animals. Here we replicate our past finding that atomoxetine improves score on the crGT, with the effect size being nearly 1.5 times greater following intra-lOFC administration in males than that previously observed following i.p. atomoxetine (Chernoff et al. 2021). This suggests the lOFC as a main locus at which atomoxetine acts in the male brain to improve cue-guided decision making. The adaptive gain theory offers a potential mechanistic framework through which local NET blockade produced these results. The theory posits that burst-like phasic noradrenaline release from the LC, which can be evoked by salient and motivationally relevant stimuli (Aston-Jones et al. 1994; Bouret & Richmond 2015; Bouret & Sara 2004), promotes fixed attention and persistent engagement in the current behavioural strategy (Aston-Jones & Cohen 2005). Conversely, as a behaviour becomes less profitable, tonic LC noradrenaline release ramps, which attenuates stimulus-triggered phasic responses and encourages exploration of other potentially lucrative options (Aston-Jones & Cohen 2005; Usher et al. 1999). As such, artificially enhancing synaptic noradrenergic tone with a NET blocker like atomoxetine could conceivably mimic the synaptic environment induced by heightened tonic LC activity, blunting the behavioural impact of cue-evoked phasic bursts and shifting behaviour toward sampling from more advantageous choices on the crGT. This interpretation is consistent with a more recent theory proposing that, specifically, tonic noradrenaline within the OFC is important for managing internal representations of task contingencies and precipitating appropriate changes in behavioural strategies to maintain performance (Sadacca et al. 2017).

Local atomoxetine may also improve crGT performance by increasing the signal-to-noise ratio in the lOFC. The noradrenaline system tunes cortical information processing by enhancing neuronal responses to relevant stimuli (signal) and diminishing responsivity to erroneous or distracting stimuli (noise) (Berridge & Waterhouse 2003; Gamo et al. 2010; Hasselmo et al. 1997; Salgado et al. 2012). Given that the lOFC responds to and encodes punishment (O’Doherty et al. 2001; Turner et al. 2021), increasing synaptic noradrenaline levels in the lOFC could potentially enhance the resultant “punishment” signal following losses on the crGT and increase behavioural sensitivity to time-out punishments, driving preference away from the risky options that deliver longer and more frequent time-outs. This interpretation is strongly supported by our trial-by-trial analyses which reveal that the improvement in score following lOFC atomoxetine in males is accompanied by an increased tendency to switch to optimal options after experiencing a risky loss. Intra-lOFC atomoxetine did not significantly change switching behaviour following wins, suggesting that the drug may not be improving crGT performance by enhancing sensitivity to reward but instead by increasing the behavioural impact of punishments. This explanation is in line with recent work fitting a reinforcement learning model to rGT data, suggesting that a relative insensitivity to punishment, instead of alterations in reward learning, drives risk taking in the presence of win cues (Langdon et al. 2019).

A higher signal-to-noise ratio could conversely promote safer decisions by boosting orbitofrontal “value” signals and/or regulating internal representations of task structure (Padoa-Schioppa & Conen 2017; Zhou et al. 2021) such that subjects increasingly favor optimal options that yield greater overall gain across a session. In further support of this hypothesis, value signals in the OFC selectively reflect the value of an attended target (Xie et al. 2018), and noradrenergic signaling importantly guides attentional allocation. Higher noradrenergic tone promotes broader, “scanning” attention (Aston-Jones & Cohen 2005; Milstein et al. 2010; Valentino & Van Bockstaele 2008), and as such, intra-lOFC atomoxetine could encourage subjects to attend to all crGT options more equally throughout the session. Therefore, value signals for each option would be more accurately represented in the decision making landscape, which could promote preference for safe crGT options that are, objectively, more profitable.

However, the inability of intra-lOFC atomoxetine to alter decision making in females is in stark contrast to our previous findings that systemic atomoxetine improves choice score on the crGT in all subjects, irrespective of sex (Chernoff et al. 2021). This may speak to possible sex differences in lOFC contribution to the neural processes underlying risk taking. In support of this conclusion, human data indicate potential sex differences in decision-related lOFC recruitment, whereby men demonstrated significant lOFC activation during performance of the Iowa Gambling Task (IGT), the human decision making paradigm from which the rGT was adapted, while women did not (Bolla et al. 2004). A preclinical study also found significant sex differences in on-task lOFC activation in rats during a probabilistic decision making task, yet the directionality of this sex difference was not indicated (van Hasselt et al. 2012).

While the data implicate the lOFC as an important mediator of atomoxetine’s benefits on decision making, at least in males, we find that intra-lOFC guanfacine was unable to sway risk preference on the crGT. This is inconsistent with our past findings, whereby systemically administered guanfacine improved decision making (Chernoff et al. 2021), suggesting that guanfacine does not reduce risk preference through action in the lOFC. In our previous experiment, however, a precise behavioural phenotype (i.e., intermediate baseline choice profiles) and dose were required for guanfacine to improve crGT score (Chernoff et al. 2021), and these may not have been represented adequately in the current smaller cohort and narrower dose range.

The disparity between our findings here and those from prior systemic experiments could also be due to the differential route of administration. When injected intraperitoneally, guanfacine acts through autoreceptors to inhibit LC cell firing and reduce noradrenaline release (Callado & Stamford 1999; Engberg & Eriksson 1991), an effect which can be replicated by direct infusion of guanfacine onto LC cell bodies (Okada et al. 2019). Conversely, when administered directly into the prefrontal cortex, guanfacine does not alter synaptic catecholamine levels (Okada et al. 2019). Multiple experiments testing working memory, attention, and impulsive choice indicate that the cognitive benefits of guanfacine are not driven by autoreceptor-mediated mechanisms, but instead by action at postsynaptic α2A receptors in the prefrontal cortex (A. Arnsten et al. 1988; Nishitomi et al. 2018; Ramos & Arnsten 2007; Wang et al. 2007). Our current results could suggest that prefrontal α2A receptor activation may not be as critical for neural processes underlying cost–benefit decision making as it is for other prefrontal functions, although this requires further replication.

Whereas intra-lOFC infusions of atomoxetine led to changes in decision making but not motor impulsivity, intra-PrL infusions of both noradrenergic drugs altered motor impulsivity but not decision making score. This functional double dissociation is consistent with anatomical studies revealing separate, direct projections from the LC to various prefrontal regions including the lOFC and PrL (Chandler et al. 2013, 2014; Robertson et al. 2013). The dissociable consequences of intra-lOFC and intra-PrL noradrenergic manipulations are also in line with the separable roles of these frontal regions in action-outcome evaluation and impulse control, respectively (Izquierdo 2017; Moschak & Carelli 2021). However, while our manipulations implicate noradrenaline signaling in the PrL, but not lOFC, in impulse control, other work using a 2-choice reaction time task in mice suggests a more important role of LC-lOFC versus LC-PrL projections in response inhibition (Bari et al. 2020). The latter specifically manipulated LC activity using optogenetics, rather than altering noradrenergic transmission via pharmacology as used here. Given the heterogeneous nature of motor impulsivity (Caswell et al. 2015), such discrepancies could reflect differences in the cognitive processes underlying premature responding when attentional load is low (current task) vs high (Bari et al. 2020). The current study also targeted slightly more lateral portions of the OFC, and different OFC subregions can exert disparate, and even antagonistic, effects on behaviour (Izquierdo 2017; Mar et al. 2011). Further work will clearly be required to resolve the functional contributions of these distinct noradrenergic projections.

Interestingly, while both atomoxetine and guanfacine reduced motor impulsivity on the crGT and other behavioural paradigms when administered systemically (Chernoff et al. 2021; Robinson et al. 2008), the high dose of intra-PrL atomoxetine reduced impulsivity in risk preferring rats, yet both doses of intra-PrL guanfacine increased premature responding in all subjects. The anti-impulsivity benefits conferred by systemic α2A agonism may therefore not be driven by direct activation of PrL α2A receptors, and guanfacine acting in the PrL might paradoxically oppose such α2-mediated benefits. Given that the α2A receptor is G_i_-coupled and generally inhibits prefrontal neuronal transmission (Ji et al. 2008), local guanfacine acting in the PrL might therefore enhance motor impulsivity largely through its inhibitory influence on prelimbic principal neurons. Reversible inactivation of the PrL impaired the ability of animals to wait for a target stimulus (Narayanan et al. 2006), increasing premature responding similar to the effects of intra-PrL guanfacine reported here. Additionally, PrL lesions or inactivation also lead to non-specific behavioural activation (Brito & Brito 1990; Jonkman et al. 2009), suggesting that the PrL might play a more general role in orchestrating behavioural inhibition. However, we did not observe any general signs of increased motor output following intra-PrL infusions, as response latencies, omissions, and trials completed were all unaffected. Guanfacine’s actions therefore appear more nuanced than expected from a simple global inhibition of PrL activity.

The selective impact of intra-PrL atomoxetine on premature responding in risk preferring rats could indicate potential differences in noradrenergic regulation of behaviour between subjects that are vulnerable versus resistant to developing risky strategies in the presence of win-paired cues. While, to our knowledge, there is currently no work defining underlying alterations in the noradrenaline system between risky and safe decision makers, other work indicates differences in NET availability (Hesse et al. 2017), autoreceptor-mediated inhibition of prefrontal noradrenaline release (Russell 2002), striatal noradrenaline levels (Moreno et al. 2010), and behavioural sensitivity to noradrenergic drugs (Ansquer et al. 2014) between high and low impulsive humans and rodents. These findings suggest that there may be biobehavioural differences in noradrenaline system function between individuals with low or high trait impulsivity. Previous work from our lab indicates that motor impulsivity and decision making are not wholly independent behavioural constructs, as premature response rates negatively correlate with decision making scores on the uncued rGT (Barrus et al. 2015) and individual differences in impulsivity account for ~ 7% of the variability in choice score on the crGT (Hynes et al. 2021). Considering this relationship between risk preference and impulsivity, risk preferring rats may exhibit some of the aforementioned alterations in noradrenaline function characteristic of highly impulsive individuals, which could help explain why risk preferring animals were uniquely sensitive to intra-PrL atomoxetine over their optimal performing counterparts.

While atomoxetine and guanfacine demonstrate incredibly selective affinities at their respective noradrenergic targets, NET, and α2A adrenergic receptors (Devedjian et al. 1994; Gehlert et al. 1995; Uhlén & Wikberg 1991), we cannot discount potential effects on the dopamine system. Given the relative dearth of dopamine transporter (DAT) in the prefrontal cortex, NET plays an important role in prefrontal dopamine clearance (Bymaster et al. 2002; Yamamoto & Novotney 1998). However, more recent evidence demonstrates that DAT-mediated dopamine uptake was nearly twice the magnitude in the OFC than in the mPFC (Yates et al. 2016), such that atomoxetine-induced increases in dopamine may be less of a concern for our lOFC pharmacology data. Dopamine can also be co-released by noradrenergic terminals in the PFC (Devoto et al. 2005), and α2 adrenergic receptors are expressed on non-noradrenergic neurons in the PFC, including dopamine neurons (Castelli et al. 2016). Accordingly, noradrenaline can modulate prefrontal dopamine release (Shinohara et al. 2020). In efforts to circumvent potential dopaminergic confounds, we administered a notably low dose of guanfacine (0.005 µg). Doses of this magnitude are purported to more selectively target the noradrenergic system (Bari et al. 2011), as lower concentrations of guanfacine should more readily bind to the denser population of α2A receptors on noradrenergic terminals versus the more sparsely expressed α2A receptors on other types of prefrontal neurons (Castelli et al. 2016). We observed identical behavioural effects of intra-PrL guanfacine at both the low and high dose (0.005 µg and 3.0 µg), suggesting that, even if the higher dose had off-target effects on dopamine release, this was not behaviourally relevant with respect to impulsivity on the crGT.

Here we illustrate a double dissociation of prefrontal noradrenergic contributions to risk taking and impulsivity across the lOFC and PrL respectively. We highlight a novel sex difference, such that noradrenergic tone in the lOFC critically guides cost benefit decision making in males but not females. Moreover, our trial-by-trial analyses support that noradrenaline signaling in the lOFC may enhance behavioural sensitivity to punishment, at least in males. Our data also indicate potential differences in noradrenaline function between rats that develop risky versus optimal decision making profiles in the presence of risk-promoting win cues. These data deepen our understanding of the neural mechanisms behind noradrenergic regulation of risky and impulsive behaviours, providing important insight into the means by which noradrenergic medications such as atomoxetine and guanfacine could ameliorate maladaptive behaviours in gambling disorder and related psychopathologies.

### Supplementary information

Below is the link to the electronic supplementary material.

Supplementary file1 (PDF 2243 KB) Figure S1. *Baseline sex differences – other behavioural variables. *A) Collection latency and C) choice latency did not differ between males and females in the lOFC cohort, yet PrL females were quicker to B) collect reward and D) choose an option than PrL males. E) lOFC females completed more trials than lOFC males, yet F) PrL males finished more trials than PrL females. G,H) There were no sex differences in omitted trials in either cohort. # between subjects group difference.

Supplementary file2 (PDF 2257 KB) Figure S2. *Null behavioural effects of intra-lOFC drug – other variables. *A-H) Neither atomoxetine nor guanfacine significantly influenced latency to collect reward, latency to make a choice, completed trials, or omitted trials when infused into the lOFC.

Supplementary file3 (PDF 2251 KB) Figure S3. *Intra-PrL drug infusions – null interactions. *A) Regarding premature responses, there was no significant interaction with dose and impulsivity level following atomoxetine infusions into the PrL. This helps support that our selective effect in risk preferring animals was not driven by baseline impulsivity. B) It appears as it the significant dose × sex × risk preference interaction for decision making score following intra-PrL guanfacine infusions was driven by subthreshold reductions in score in risk preferring males only, which did not reach statistical significance on any follow up tests.

Supplementary file4 (PDF 2257 KB) Figure S4. *Null behavioural effects of intra-PrL drug infusions on other behavioural variables. *A-H) Following infusion into the PrL cortex, neither atomoxetine nor guanfacine affected collection latency, choice latency, completed trials, or omissions.

## Data Availability

Data are available from the corresponding authors upon reasonable request.
