# Supplementary figures and images for: Noradrenergic regulation of cue-guided decision making and impulsivity is doubly dissociable across frontal brain regions
Source: Psychopharmacology (Berl). 2023 Nov 25;241(4):767–83. doi: 10.1007/s00213-023-06508-2 (PMC10927866; doi:10.1007/s00213-023-06508-2)

Supplementary Figure 1: Baseline sex differences - other behavioural variables

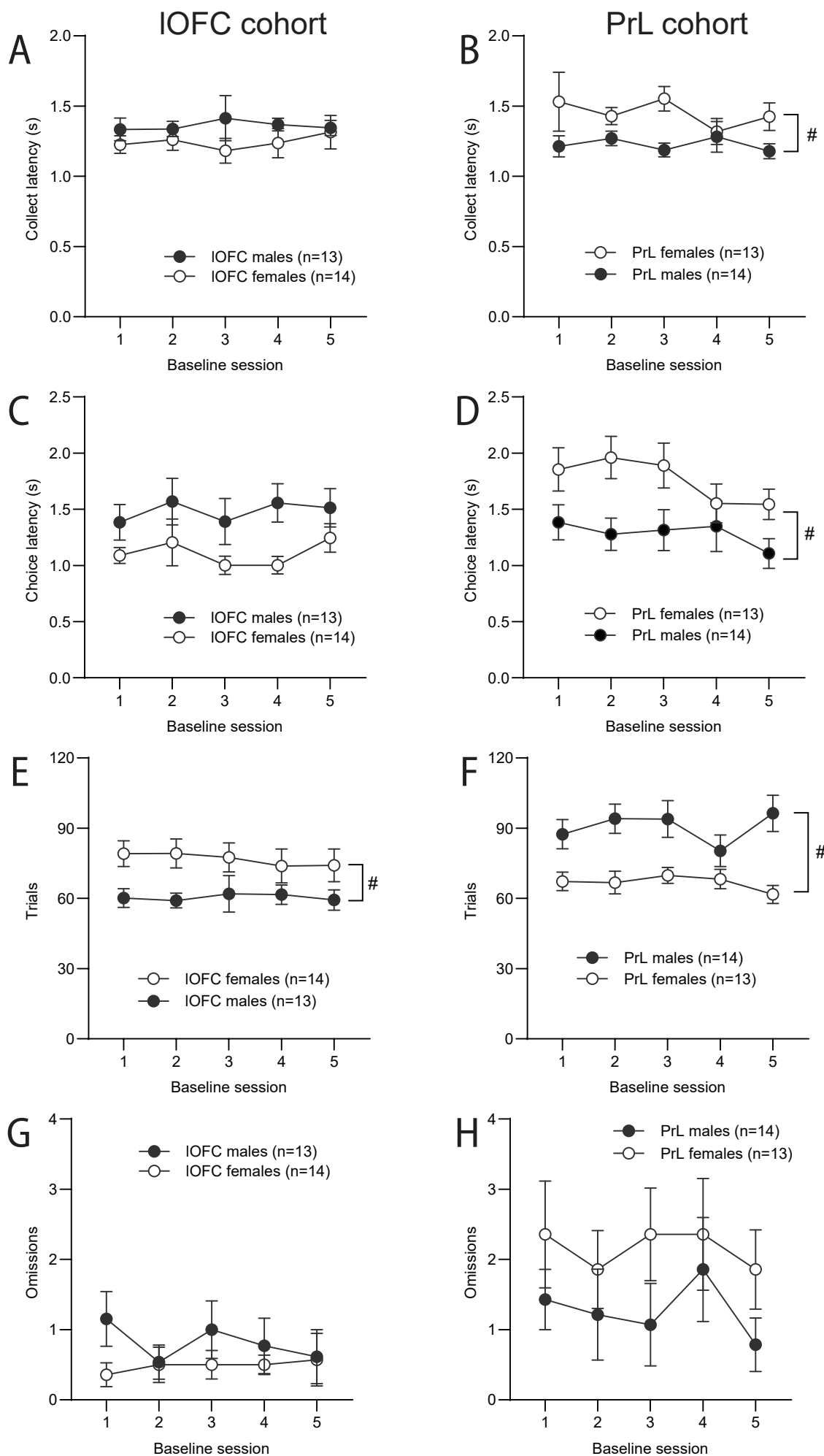

Supplement: Supplementary file 1 — Supplementary file1 (PDF 2243 KB) Figure S1. Baseline sex differences – other behavioural variables. A) Collection latency and C) choice latency did not differ between males and females in the lOFC cohort, yet PrL females were quicker to B) collect reward and D) choose an option than PrL males. E) lOFC females completed more trials than lOFC males, yet F) PrL males finished more trials than PrL females. G,H) There were no sex differences in omitted trials in either cohort. # between subjects group difference. [file 213_2023_6508_MOESM1_ESM.pdf]

Supplementary Figure 3: Intra-PrL drug infusions – null interactions

Atomoxetine

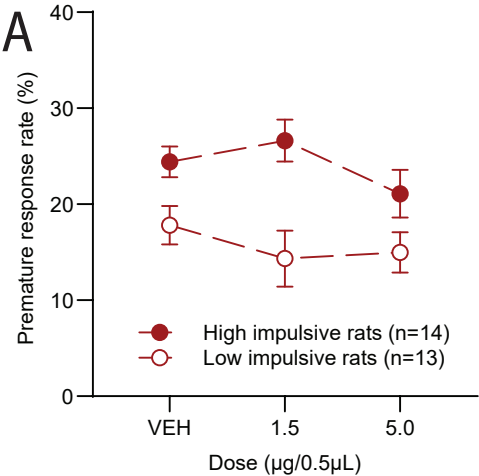

Guanfacine

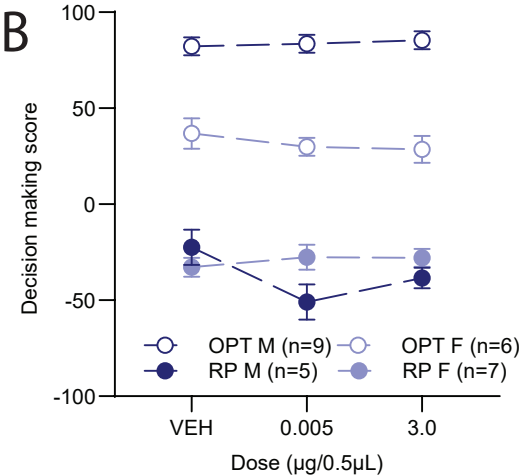

Supplement: Supplementary file 3 — Supplementary file3 (PDF 2251 KB) Figure S3. Intra-PrL drug infusions – null interactions. A) Regarding premature responses, there was no significant interaction with dose and impulsivity level following atomoxetine infusions into the PrL. This helps support that our selective effect in risk preferring animals was not driven by baseline impulsivity. B) It appears as it the significant dose × sex × risk preference interaction for decision making score following intra-PrL guanfacine infusions was driven by subthreshold reductions in score in risk preferring males only, which did not reach statistical significance on any follow up tests. [file 213_2023_6508_MOESM3_ESM.pdf]

Supplementary Figure 4: Intra-PrL drug infusions – other behavioural variables

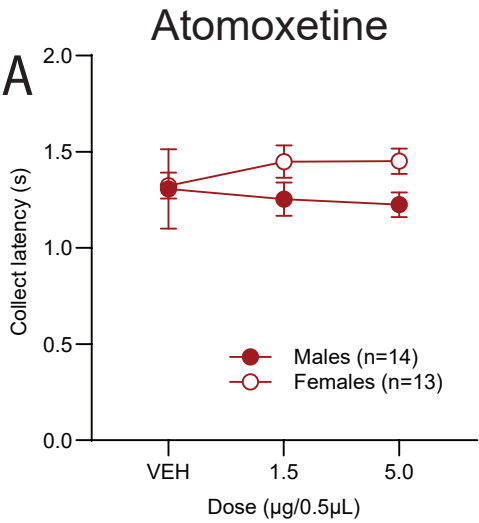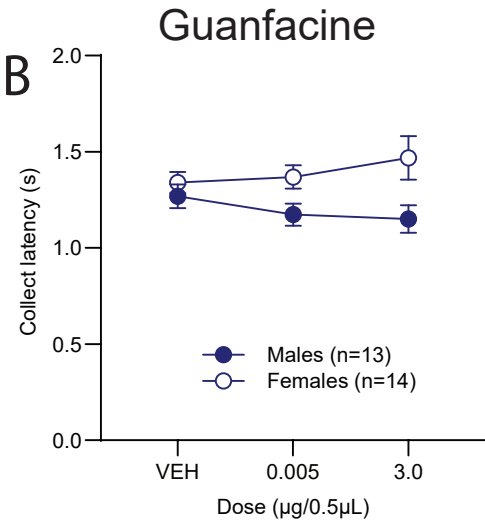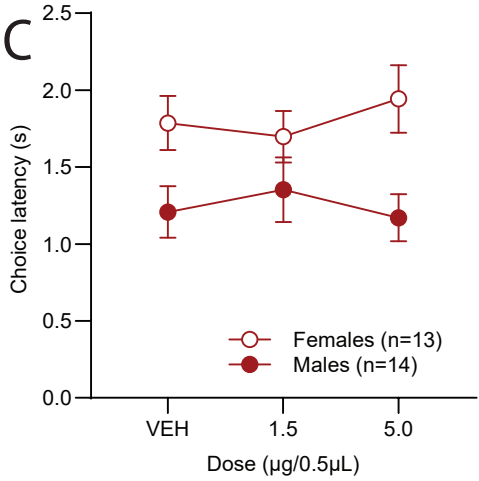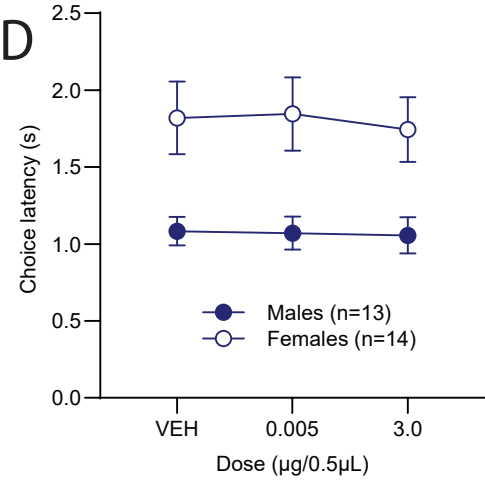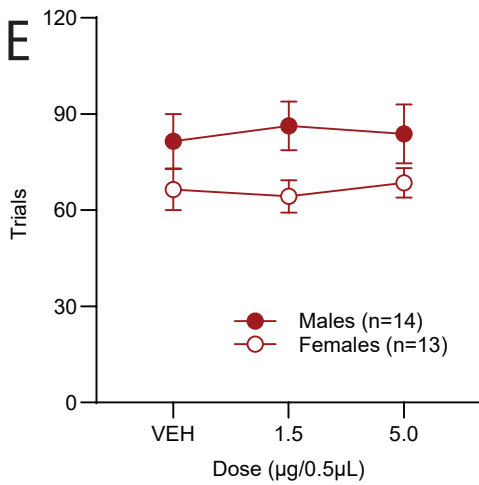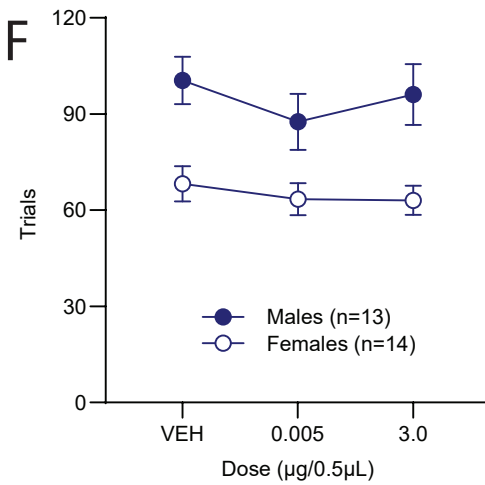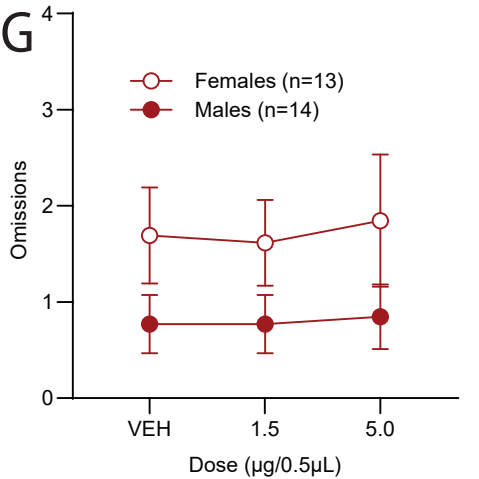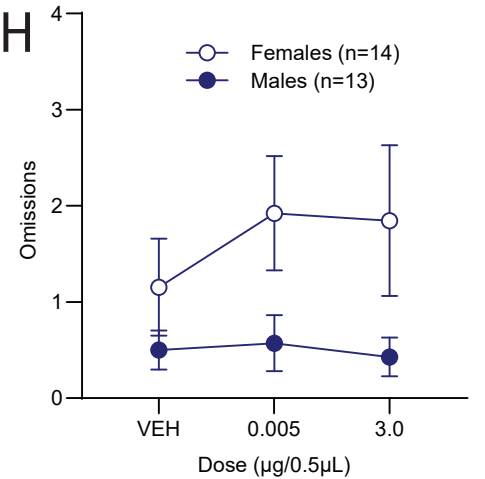

Supplement: Supplementary file 4 — Supplementary file4 (PDF 2257 KB) Figure S4. Null behavioural effects of intra-PrL drug infusions on other behavioural variables. A-H) Following infusion into the PrL cortex, neither atomoxetine nor guanfacine affected collection latency, choice latency, completed trials, or omissions. [file 213_2023_6508_MOESM4_ESM.pdf]
