# Supplementary material for: Noradrenergic regulation of cue-guided decision making and impulsivity is doubly dissociable across frontal brain regions
Source: Psychopharmacology (Berl). 2023 Nov 25;241(4):767–83. doi: 10.1007/s00213-023-06508-2 (PMC10927866; doi:10.1007/s00213-023-06508-2)

Supplementary Figure 2: Intra-lOFC drug infusions - other behavioural variables.

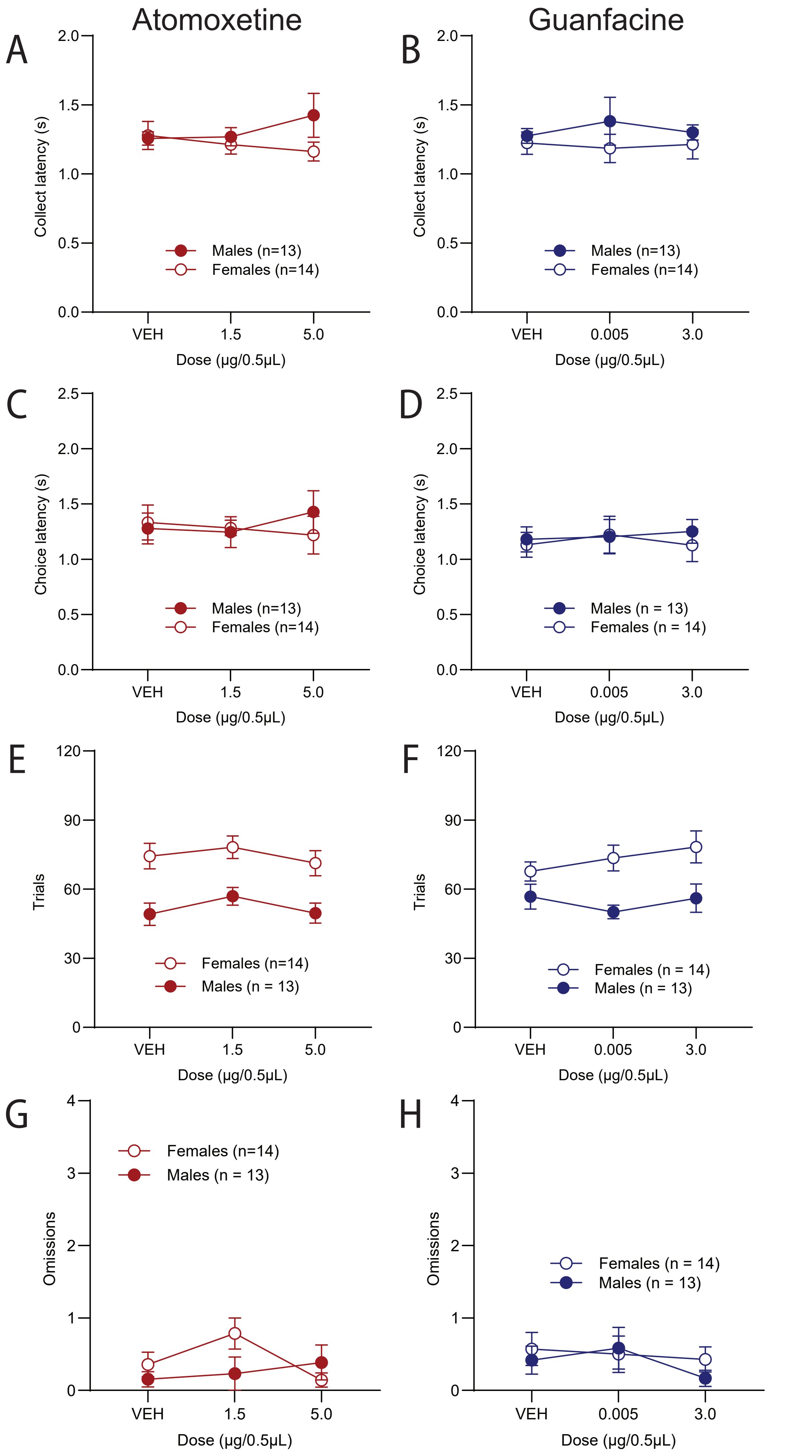

Supplement: Supplementary file 2 — Supplementary file2 (PDF 2257 KB) Figure S2. Null behavioural effects of intra-lOFC drug – other variables. A-H) Neither atomoxetine nor guanfacine significantly influenced latency to collect reward, latency to make a choice, completed trials, or omitted trials when infused into the lOFC. [file 213_2023_6508_MOESM2_ESM.pdf]
